# Attachment-based parent–adolescent interaction linked to visual attention and autonomic arousal to distress and comfort stimuli

**DOI:** 10.1186/s40359-022-00821-9

**Published:** 2022-05-02

**Authors:** Marie Schneider, Ingrid Obsuth, Monika Szymanska, Julie Mathieu, Sylvie Nezelof, Karlen Lyons-Ruth, Lauriane Vulliez-Coady

**Affiliations:** 1 Child and Adolescent Psychiatry Department, Franche Comté University, Besançon, France; 2 Department of Clinical Psychology, University of Edinburgh, Edinburgh, UK; 3 Science and Technology Department, Laboratory of Integrative and Clinical Neuroscience, University of Burgundy Franche‐Comté, Besançon, France; 4 Department of Psychiatry, Harvard Medical School, Boston, USA

**Keywords:** Adolescents, Attachment, Parent–child relationship, Emotion regulation, Eye-tracking

## Abstract

In infancy and in the early years of life, emotion regulation and attachment relationships with parents are tightly intertwined. However, whether this link persists into adolescence has not yet been established and requires exploration. This pilot study utilizes an experimental design to assess the patterns of parent–adolescent interactions that are hypothesised to be related to two specific aspects of adolescents’ emotion regulation, namely: visual attention and autonomic arousal to distress and comfort stimuli. Two innovative and ecologically valid methodologies were utilized to assess (a) patterns of attachment-based parent–adolescent interactions among 39 adolescent–parent dyads from the general population, using the Goal-corrected Partnership in Adolescence Coding System (Lyons-Ruth et al. Goal corrected partnership in adolescence coding system (GPACS), 2005) applied to a conflict discussion task; (b) the two aspects of adolescent emotion regulation were assessed with the Visual/Autonomic Regulation of Emotions Assessment (VAREA) (Vulliez-Coady et al. Visual/Autonomic Regulation of Emotions Assessment, VAREA) paradigm, an attachment-related, emotionally arousing experimental procedure, using a distress-then-comfort paradigm, in conjunction to an eye-tracker synchronized with a physiological device that measured gaze and skin conductance response, (SCR), or emotional reactivity. In line with research in infancy, as predicted, markers of secure parent–adolescent interaction were linked to higher amplitude of SCR for distress and comfort pictures, and with longer attention to comfort pictures. On the other hand, parental role-confusion was associated with less time spent on comfort pictures by the adolescent. Overall, this pilot study suggests that interventions supporting collaborative communication between adolescents and their parents, as well as working to reduce parental role-confusion, may improve adaptive adolescent emotion regulation as assessed via physiological measures.

## Introduction

Emotion regulation refers to a multifaceted phenomenon that encompasses changes in the quality, intensity, duration, and latency of emotional reaction and expression in the service of adaptation [1]. It is a sophisticated process relying on the temporary synchronization of physiological, behavioral, and cognitive components (attention processes, encoding emotional cues, selecting an adaptative mode for expressing emotion) [1, 2]). Yet, the majority of existing measures of emotion regulation have focused on assessing self-reported cognitive elements of one’s responses to emotional experiences [3]. Fewer studies have examined the behavioral and physiological aspects of emotion regulation [4]. In this study, we focus on two aspects of emotion regulation in response to pictures of distress and comfort, visual attention and skin conductance reactivity (autonomic arousal) assessed at the same time. There is also evidence that the way in which individuals respond to emotional situations is shaped by interactions with attachment figures (i.e., caregivers/parents; see below). Therefore, we examine the behavioral and physiological elements of emotion regulation in relation to the quality of parent–adolescent interactions.

### Interpersonal context of development

Children’s socio-emotional development occurs in a social context. ‘Socialization of emotion’ a term coined in 1928 [5] was introduced to capture the idea that children learn to understand, express and regulate emotions in social situations. According to Eisenberg, Cumberland, and Spindrad [6], consistent with social learning theory, ‘emotion socialization’ occurs via similar processes as general socialization, that is via observation and imitations of others [7], as well as through a range of reinforcement practices [8]. Complementary to emotion socialization and social learning theory, attachment theory and research describes distinct emotion regulation strategies resulting from different patterns of caregiver-infant interactions [9, 10]. Attachment theory posits that repeated interactions with caregivers contribute to the development of a stable internalized representation, or internal working model, of the caregiver’s availability for reducing stress and providing comfort and protection in potentially threatening situations [11–13].

There is an extensive body of evidence that the security of parental working models of attachment relationships influences the behavioral emotion regulation strategies that infants and young children use in responding to stressful situations [14]. Based on the sensitivity and responsiveness of the interactions, early experiences with caregivers may lead to more balanced and adaptive secure strategies of regulation or less well-regulated and less adaptive insecure (avoidant, anxious, disorganized) strategies [14, 15]. Security of attachment in infants has also been linked to attenuated stress hormone responses (cortisol elevations) compared to insecure infants [16, 17]. Insecure strategies, in turn, have been related to less adaptive oucomes throughout childhood in several meta-analyses [18–20].

### Attachment and emotion regulation

A number of studies also have linked the security of attachment relationships to differences in expressing, inhibiting and regulating emotions [10, 21–26]. Indeed, individuals with a predominantly secure attachment have been shown to exhibit a sense of self-efficacy when dealing with distress [27] and to be able to appropriately ask for help when they need support [28].

In contrast, individuals with a more insecure-anxious attachment are more likely to hyperactivate their attention to emotional cues, which is associated with overarousal toward threats to the self and fears of abandonment [10]. On the other hand, individuals with a more insecure-avoidant attachment will utilise deactivating strategies, avoid dealing with their distress, and fail to seek out others for help [29]. Finally, individuals with disorganized attachments, whose caregivers more often exhibit hostile/punitive behaviors, parental role-confusion, and misattuned affect, are more likely to exhibit no consistent strategy for seeking comfort and modulating their distress [30].

While links between emotion regulation and attachment have been well-established in childhood and adulthood, much less is known about both attachment and emotion regulation during the crucial stage of adolescence. Therefore, in this pilot study, we examine the link between specific aspects of emotion regulation and attachment utilizing an experimental approach and a combination of attachment behavior observations and behavioral/physiological assessments of emotion regulation.

### Attachment and emotion regulation during adolescence

Adolescence is one of the most significant developmental transitions across the lifespan, incorporating extensive neurobiological, cognitive, psychological and social changes in a short amount of time [31, 32]. This period of transition to adulthood comes with major developmental milestones, including the need to navigate a complex and widening social network, negotiating first romantic relationships, and the increased need for autonomy and the exercise of more responsibility for one’s decisions and actions. It is also marked by an elevated risk for suicide and an increase in mental health disorders, including depression, borderline personality disorder, substance abuse, and eating disorders [33–35]. Thus, adolescence is being increasingly recognized as a “window” of both opportunity and risk in development. Although, emotion dysregulation, defined as “a pattern of emotional experience and/or expression that interferes with appropriate goal-directed behavior” [36] is identified as a key contributer to risk in adolescence [37–42], the literature on emotion regulation in adolescence is not consistent, possibly due to both diverse assessment methods for emotion regulation and different ages of assessment within the adolescent period. The general trend in the literature points to increasingly adaptive emotion regulation from middle adolescence to emerging adulthood. However, several factors influence this general developmental trend, including genetic factors, age, gender, environmental factors, and the particular emotion being regulated [41, 43]. One of those factors is the attachement relationship.

The attachment relationship continues to be an important influence on emotion regulation in adolescence. During childhood and adolescence, the attachment relationship evolves into a goal-corrected partnership in which the child’s security, that is, their confidence in the caregiver’s availability, is maintained by the quality of open and balanced communication in the relationship [44]. While attachment studies have mainly focused on mothers, more studies have begun to assess both mother-adolescent interaction and father-adolescent interaction, with no differences found in reciprocity, parental sensitivity and adolescent outcomes [45–47].

The concept of open and balanced communication has been most frequently operationalized in the context of a discussion of a conflict in the relationship, which has been found to be mildly stressful. The arousal-eliciting nature of this paradigm in adolescence was supported by Marceau et al. [48], who found elevations in cortisol levels in response to a conflict discussion in a sample of 217 adolescent boys and girls (M age = 13). Open and balanced communication is characterized by the extent to which the parent–adolescent dyad can discuss conflicted topics in a way that communicates each person’s goals while respectfully acknowledging the other’s point of view [49]. Importantly, balanced or collaborative exchanges in early adolescence assessed during a parent–adolescent conflict discussion predict the emergence of the adolescent’s secure attachment as assessed on the Adult Attachment Interview at age 19 [50, 51]^.^ Parental sensitivity in interaction is also associated with a secure adolescent attachment representation and with adaptive adolescent emotion regulation [52–56]. Importantly, increased maternal sensitive support during adolescence can promote a shift toward attachment security among teens who were insecurely attached as infants [57]. Sensitivity dimensions, such as maternal attunement to adolescent self-perception and high levels of relatedness during disagreements on critical issues, are also associated with increased adolescent autonomy [26, 58, 59] and exploration of independence in thought and speech [60]. Therefore, it is possible that more sensitive and collaborative interactions with caregivers in adolescence would be associated with more adaptive behavioral strategies and physiological reactivity for modulating emotional arousal in adolescence.

### Visual attention and skin conductance components of emotion regulation

Few studies to date have assessed measures of visual attention and autonomic arousal as indices of emotion regulation in relation to attachment security. Even fewer have investigated how these components of emotion regulation may differ among the insecure attachment styles [61–63]. Vandevivere et al. [64] assessed visual attention in relation to pictures of the mother’s face and unfamiliar faces among children 8- to 12-years-old and found that, compared to avoidant children, secure children had longer fixation times on their mothers’ faces than on unfamiliar women’s faces and also fixated them more often. Kammermeier et al. [65] found that attachment security was a significant predictor of longer fixation times to neutral and sad expressions while controlling for age, gender, and temperament. Visual attention to a picture of the mother’s face was also related to how long distressed children would wait before seeking their mother’s proximity [66]. Finally, when mothers’ visual attention was assessed, mothers of securely attached children presented different patterns of visual attention to their children’s faces (sad, happy, and neutral) than mothers of anxious children [67].

Regarding physiological responses assessed via skin conductance reactivity (SCR), the sparse literature is not consistent for adolescents and adults. Skin conductance responses (SCRs) reflect sympathetic nervous system (SNS) reactions to emotionally laden stimuli [68], and, as such, they represent a robust index of distress reactivity [69]. The physiological impact is reflected in the latency (lower latency, quicker reaction to stress) and the amplitude, which is defined as the peak height of the SCR (higher amplitude, the more the SNS is activated). Beijersbergen et al. [61] found no significant differences in skin conductance reactivity among secure, avoidant, and preoccupied adolescents, either during a mother–adolescent conflict interaction task (Family Interaction Task) or during the Adult Attachment Interview [70]. However, in the adult population, attachment styles have been associated with SCR. During and following a conflict with a partner, Taylor et al. [71] found that avoidant adults exhibited decreased levels of SCR, while Diamond et al. [72] found that in response to hypothetical separation from a partner, avoidant adults displayed heightened and escalating SCR.

### The current study

The current study builds on a larger study of visual and autonomic responses among 81 adolescents in a low-risk, non-clinical sample [73]. In this sample, a novel procedure was developed to assess how distress- and comfort-related pictures are processed visually and autonomically, the Visual/Autonomic Regulation of Emotions Assessment (VAREA; see Methods) [74]. VAREA consists of two phases: the first phase “distress exposure” (distress pictures) followed by the second phase “arousal reduction” (3 pictures—comfort, joy, and neutral presented together). This assessment tool is grounded in attachment research that indicates that distress activates the attachment system (i.e., support-seeking) and is attenuated when comfort is given, thus deactivating the attachment system [9, 51]. Based on this theory, visual attention was expected to be a behavioral index of the adolescents’ strategies to regulate distress and seek comfort. In addition, synchronized measurement of skin conductance response (SCR; autonomic arousal) with the eye gaze was expected to index the physiological reactivity associated with these strategies.

Consistently, in the first validation study of the VAREA [73], different patterns of visual attention and autonomic arousal were related to adolescents’ attachment as measured by an interview assessing secure, avoidant, and anxious attachment styles [75]. Secure adolescents fixated longer than avoidant adolescents on the distress pictures. Avoidant adolescents exhibited shorter first fixation than secure adolescents to distress pictures, in association with quicker autonomic response (decrease in latency of SCR)**.** During the following phase during which three pictures (comfort, joy and neutral) were presented simultaneously, gaze movements also differed according to attachment styles. Secure adolescents fixated first on joy then on comfort, and finally on neutral. Avoidant adolescents fixated on comfort pictures last and for the shortest durations. Anxious adolescents fixated on comfort pictures longer than both secure and avoidant adolescents.

However, the Attachment Style Interview, while consistent with attachment theory, has not been validated against attachment behavior patterns coded from observed adolescent–parent interactions.Therefore, it is important to assess whether visual and autonomic components of emotion regulation are also related to observed attachment-related interactions in expected ways. One validated instrument for assessing attachment-related interactions is the Goal-Corrected Partnership in Adolescence Coding System [9, 76]. The GPACs is coded during a conflict discussion between the adolescent and their parent, and includes scales for dimensions of collaborative interaction and for dimensions of controlling and disorganized interaction. The GPACS scales have been found to be significantly related to established measures of attachment security, both longitudinally in relation to infant attachment and concurrently in relation to adolescent responses to the Adult Attachment interview [77].The Adult Attachment Interview is still the ‘gold standard’ for assessing attachment patterns in adolescence, even though concerns have been raised about its validity in high-risk samples [78]. The GPACS scales have also shown both concurrent and predictive validity in relation to several measures of adolescent psychopathology, including depression, suicidality, and impulsive self-damaging behavior [25, 77, 79].

While the original version of the GPACS, which was used in this study, does not include coding scales that directly measure anxious and avoidant attachment styles [80], it does include scales that assess several aspects of secure attachment, as well as scales rating aspects of insecure and disorganized interactions, including hostility, role-confusion, and disorientation (see Methods for further details). However, no studies have yet explored how these aspects of parent–adolescent interactions on the GPACS are related to emotion regulation, specifically to the adolescent’s visual and autonomic responses to distress and comfort-related stimuli.

The aim of this exploratory pilot study was to further assess the validity of the VAREA in relation to observed attachment interactions measured with the GPACS. The study builds on and extends prior findings on the VAREA by Szymanska that found different patterns of gaze and SCR to secure and insecure aspects of attachment on an interview measure of attachment [73]. Based on those findings, we hypothesized that secure aspects of parent–adolescent interaction and insecure/disorganized aspects of parent–adolescent interaction would be associated with different patterning of adolescent gaze and SCR responses to distress and comfort stimuli. Our hypotheses are the following: (1) to the distress picture, aspects of secure parent–adolescent interaction will be associated with higher amplitude and longer latency of SCR and longer fixation time, and to comfort pictures, with higher amplitude of SCR and longer gaze fixation time to comfort picture with a first gaze orientation on comfort pictures. This pattern of response is congruent with a secure adolescent being more open to both negative and positive feelings, as well as being more open to seeking comfort under stress. There is little previous work on which to base hypotheses about how parental hostility, role-confusion, or disorientation might relate to behavioral and autonomic responses on the VAREA. Based on Syzmanska et al.’s [73] findings regarding avoidant adolescents, we tentatively hypothesized that (2) to the distress picture, negative parental dimensions of hostility, role confusion, and disorientation would be associated with the adolescent’s shorter latency of SCR and shorter gaze fixation time, and to the comfort pictures, with longer gaze entry time and shorter fixation time on comfort pictures. This pattern would be consistent with rapid recognition of the arousing stimuli, followed by moving attention away from distress stimuli and avoiding comfort stimuli.

## Materials and methods

### Participants

The sample consisted of 39 adolescents (n = 32, 82.05% girls) with one of their parents (33 mothers, 6 fathers). Adolescents were recruited from the general population in three different secondary schools in Besançon, France, and ranged from age 13 to 18 years (M = 15.31, SD = 1). All participants had normal or corrected-to-normal vision. A psychiatrist evaluated each participant’s eligibility during the first visit at school. Adolescents suffering from psychiatric disorders were not included and were referred to a consultant psychiatrist. Of the 83 screened potential participants, 81 were invited to participate and 42 adolescents and their parents agreed to participate. Three dyads were excluded from our analyses due to technical difficulties (n = 3), yielding 39 subjects for the current analyses. Written informed consent was given by the adolescents and their parents. Socio-economic status was typical of a normative risk community sample: 7.69% (n = 3) of families earned less than 1500 Euros per month, 66.67% (n = 26) of families earned between 1501 and 3999 Euros per month, and 25.64% (n = 10) families earned more than 4000 Euros per month.

### Procedure

Two weeks following the screening session, during a 2-h laboratory session, the adolescents and their parent were first taken to separate rooms. Adolescents participated in the VAREA assessment (see below). Before rejoining their parent, the adolescents were asked about topics that they viewed as sources of disagreement in the relationship with the parent, and the adolescent audio-recorded a statement of their view of the topic of conflict with the parent. The parents completed a socio-economic questionnaire. Parent and adolescent were then reunited for a 5-min unstructured meeting, followed by the playing of the taped adolescent statement about a conflict and a 10-min discussion of the topic of disagreement. All interactions were videotaped and coded by the two trained raters, using the Goal-Corrected Partnership in Adolescence Coding Scale (GPACS) [80].

Each adolescent and each parent received a voucher of 20 euros for participation. This study was conducted in accordance with the principles of the Helsinki Declaration, the French Agency for the Safety of Health products, and received approval from the local ethics committee, Committee for the Protection of Persons (CPP EST-II, number 2012-A01545-38). The study was registered with the Clinical Trials.gov under the number NCT02851810.

### Assessments

#### Coding of adolescent–parent interaction

Adolescent–parent interaction was assessed using the Goal-Corrected Partnership in Adolescence Coding Scales (GPACS) [80], applied to a videotaped interaction in which the parent and adolescent discussed a topic of conflict between them. The GPACS includes the rating of each videotape on ten five-point scales. Two scales focus on the dyad (collaborative communication and warmth) and provide an assessment of the cooperative, reciprocal and balanced nature of the dyad. The warmth scale assesses the expression of care, valuing statements, and positive regard shared between the parent and adolescent. Four scales focus on the adolescent’s behavior (respectful spontaneity, hostile/punitive, caregiving/role-confused and odd/disoriented behaviors). Four other scales focus on the parent’s behavior (validation of the adolescent’s voice, hostile/punitive, role-confused and odd/disoriented behaviors). Specifically, the adolescent caregiving/role-confused behavior scale assesses the extent to which the adolescent attempts to manage or take care of the parent or modulate the parent’s behavior (e.g., offering guidance; defusing tension with overbright, entertaining behavior). The adolescent hostile/punitive behavior scale assesses the extent to which the adolescent behaves in a hostile, punitive, or devaluing way toward the parent. The adolescent odd/disoriented behavior scale taps the extent to which the adolescent engages in odd, out-of-context, or disoriented behaviors, which may seem disjointed, startling, or inexplicable to an observer. The remaining four scales rate the behavior of the parent. The scale for parent’s validation of adolescent’s voice rates the degree to which the parent supports the adolescent’s exploration of thoughts and feelings related to the conflict. The scale for parental hostile/ punitive behavior is parallel to the adolescent scale described above, as is the parental scale for odd/disoriented behavior. The parental role-confusion scale assesses the extent to which the parent fails to assume a parental stance by failing to structure the interaction, failing to contribute to the task goals (discuss the conflict), remaining excessively self-focused (e.g., prioritizing his/her own needs over the needs of the adolescent) or treating the adolescent like a peer or a romantic partner.

A factor analysis on the GPACs scales [77] found that four scales contribute to an underlying construct of attachment security—collaborative communication, warmth, validation of the adolescent’s voice, and adolescent’s respectful spontaneity. Therefore, these scales are interpreted in this study as indicators of secure attachment. The remaining six scales tapped three aspects of insecure/disorganised attachment, namely role-confused interaction, hostie/punitive interaction, and disoriented interaction. Given that this is the first study to explore the link between these insecure/disorganized aspects of interaction and physiological aspects of emotion regulation, all six scales were utilised in the analyses.

The GPACS has shown longitudinal validity in relation to infant attachment classification, concurrent validity in relation to attachment classification on the AAI at age 19, and concurrent validity in relation to to parent and adolescent self-reports of role-confusion [77]. The GPACS has also shown construct validity in relation to multiple aspects of adolescent maladaptation and psychopathology [25, 77, 79, 81, 82]. To assess reliability, two independent raters coded 15 randomly selected tapes from the current sample. Reliability was good on all scales, ICCs = 0.82—0.95.

#### Assessment of Visual/ Autonomic Regulation of Emotions (VAREA)

**Fig. 1:**
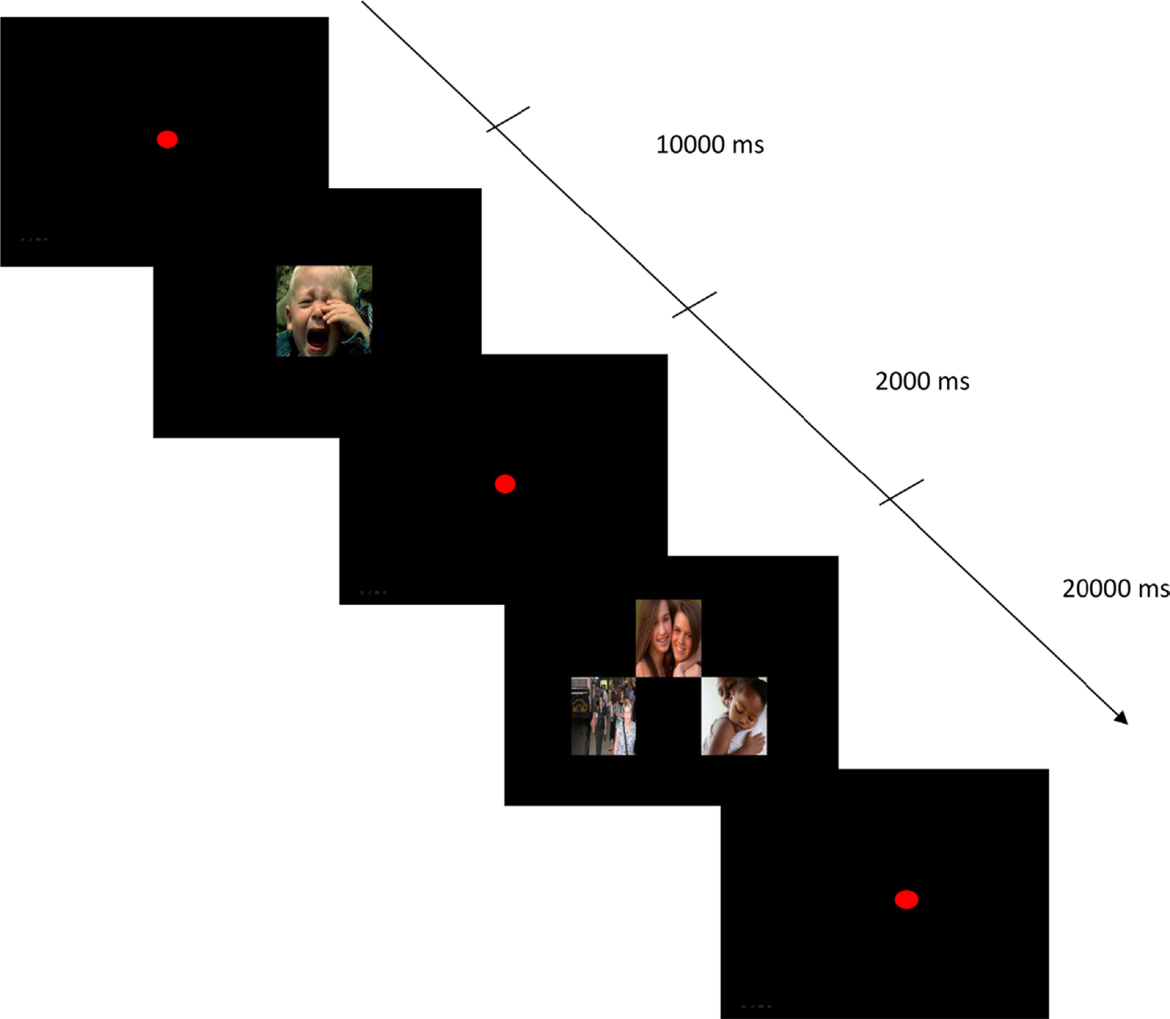
Sample of stimulus displays:Distress pictures followed by comfort, joy, neutral pictures (from the Besancon Affective Picture Set-Adolescents (BAPS-Ado) [83]

The adolescent’s visual and autonomic responses to pictures of distress and comfort were assessed using the Visual/Autonomic Regulation of Emotions Assessment (VAREA) [73, 74]. Adolescents were exposed to a series of pictures from the Besancon Affective Picture Set-Adolescents (BAPS-Ado)[83], representing distress, comfort, joy, and a neutral state. Pictures of distress represented faces expressing sadness, anguish, or scenes of loss and separation. Comfort pictures represented scenarios of a parent comforting an infant or an adolescent, or an adult comforting an adult, after an episode of distress. Pictures of joy represented joyful moments between parent and child, adults or peers. Finally, neutral scenes represented persons walking along a street or in the subway without interaction. Pictures were distributed in a series of 20 blocks presented randomly. The picture sequences were organized in two phases, with the theoretical rationale that a distress picture would be arousing, or activate the attachment system, while a comfort picture, and to a lesser extent, joyful or neutral pictures, would reduce arousal, or deactivate the attachment system. During the first phase “distress exposure”, a distress picture was presented for 10 s. During the second phase” arousal reduction”, three pictures (comfort, joy, and neutral) were presented together for 20 s (Fig. 1). VAREA investigates visual attention (fixation and entry time), which taps the aspects of ER consistent with behavioral responses/strategies to a distress and comfort situations, and skin conductance responses (amplitude and latency), which reflect the aspects of ER consistent with physiologic reactions to emotionally laden stimuli, exclusively modulated by the sympathetic nervous system [68].

#### Visual attention

Visual attention was measured by the assessment of eye movement. Eye movement is a well-known biomarker whose variations are related to psychological stress and emotional distress [64, 84, 85]. Eye movements were recorded using the Remote Eye-Tracking Device at a frequency of 250 Hz (RED 500, SMI®, Teltow, Germany, www.smivision.com). Details relating to the device are presented in the previously published protocol [86]. The areas of interest (AOI) were divided into four categories (distress, comfort, joy and neutral), with each one analyzed separately. Fixations were defined as gaze fixations of at least 80 ms on 100 pixels [87]. The following dependent variables were analyzed with the BeGaze software. During the first phase, when the distress picture was presented alone, only fixation time (ms) was measured. During the second phase “arousal reduction”, when three pictures were presented together (comfort, joy, and neutral), two variables were measured: fixation time and entry time (ms).

Fixation time represents how long the participant visually explores each picture in milliseconds (ms) and was calculated by averaging all fixation times per trial. This measure corresponds to the average sum of durations from all fixations and saccades that hit the AOI [88]. It reflects engagement patterns and can be related to an increase/decrease in the salience or visual attractive power of the picture [61, 67, 84]. Entry time reflects the time needed in milliseconds (ms) to detect emotional visual stimuli and indicates the first gaze orientation toward each of the three pictures [89], giving an indication of salience and preference. Entry time is the average time from stimulus onset to the first fixation on the AOI in milliseconds per trial [87].

#### Autonomic arousal

We measured autonomic arousal via skin conductance responses (SCR). SCRs reflect the aspects of ER consistent with physiologic reactions to emotionally laden stimuli, exclusively modulated by the sympathetic nervous system [68], and represent a robust index of distress reactivity [69]. The physiological impact can be analyzed with the latency (the lower, the quicker reaction to stress) and the amplitude (the higher, the more the SNA is activated). During the visualisation of pictures, SCRs were collected with a BIOPAC MP36 acquisition system and analyzed with the AcqKnowledge 4.3 software. The SCR was recorded with two electrodes (BIOPAC, Model EL 507) placed on the second phalanges of the index and middle fingers of the non-dominant hand. The data were then digitized at 1000 samples per second, with a gain of 1000. Amplitude and latency of SCR were analyzed. The SCR amplitude fluctuation is the peak height of the SCR minus the value of the SCR level at the time that the SRC response began. It was defined as an unambiguous increase with respect to each pretarget stimulus baseline, occurring 0.01–6.0 s after the target stimulus, based on timings used by Thompson [90],and Siller [91].In humans, the amplitude of SCR is related to the level of arousal elicited by visual stimuli with either positive or negative emotional valence. The SCR latency was defined as the separation between the stimulus event and SCR’s first deflection from baseline.

In summary, in relation to distress pictures, VAREA yields one gaze parameter—fixation time, and two autonomic arousal parameters—SCR amplitude and latency. In relation to comfort pictures, VAREA yields two gaze parameters—fixation time and entry time, and two autonomic arousal parameters—SCR amplitude and latency. These are the measures analyzed in relation to attachment dimensions on the GPACS.

### Statistical data analysis

Statistical analyses were performed using SAS, version 9.4 (SAS institute, Cary, NC). Data were first screened for outliers. Outliers for each eye-tracking variable were deleted from the analyses, using a z-score with a threshold of mean ± 3.29. After threshold application, almost all data were retained (99.9%). As data were not normally distributed, nonparametric tests were used in all analyses. Namely, Spearman’s correlation coefficients were calculated to assess links between dimensions of adolescent–parent interaction and parameters related to gaze and autonomic arousal. For Spearman coefficient values, strength of association is as follows: very weak 0–0.19, weak for 0.2–0.39, moderate for 0.40–0.59, strong for 0.6–0.79 and very strong for 0.8–1 [92].

## Results

### Distress picture parameters in relation to parent–adolescent interaction dimensions

#### Secure parent–adolescent interaction dimensions

We hypothesized that, to the distress picture, aspects of secure parent–adolescent interaction would be associated with higher amplitude and longer latency of SCR and longer fixation time. Spearman correlation analyses indicated a significant positive correlation between SCR amplitude and all secure dyadic dimensions, including collaborative communication (*rho* = 0.52, *p* = 0.005), dyadic warmth (*rho* = 0.61, *p* = 0.0006), parental validation of the adolescent’s voice (*rho* = 0.52, *p* = 0.005), and adolescent respectful spontaneity (*rho* = 0.43, *p* = 0.03). Thus, the hypothesis that adolescents experiencing collaborative parent–adolescent interactions would show higher amplitude SCR when looking at distress pictures was supported, although the expectation of longer latency of SCR was not supported. Complete results are available in Table 1.

**Table 1:** Associations between skin conductance responses and aspects of parent–adolescent interaction in response to distress and comfort pictures

Dimensions	Skin conductance responses			
	Distress pictures phase		Comfort pictures phase	
	Amplitude	Latency	Amplitude	Latency
*Dyadic*				
Collaborative communication	0.52**	− 0.33	0.34	− 0.25
Warmth	0.62***	− 0.26	0.53**	− 0.39*
*Parent*				
Validation of the adolescent’s voice	0.52**	− 0.16	0.26	− 0.12
Hostile/punitive	− 0.19	0.34	0.09	− 0.19
Role-confusion	0.01	0.12	0.04	0.04
Odd/disorientation	− 0.09	0.29	− 0.06	− 0.11
*Adolescent*				
Respectful spontaneity	0.43*	− 0.36	0.40*	− 0.25
Hostile/punitive	− 0.22	0.25	− 0.28	0.33
Role confusion	0.07	− 0.11	0.22	0.03
Odd/disorientation	− 0.19	0.09	− 0.36	0.32

N = 39, rho statistic is shown in table

*p < 0.05; **p < 0.01; ***p < 0.001

In addition, no significant correlations were found between secure-collaborative parent–adolescent interaction dimensions and adolescent fixation time in response to the distress pictures. Thus, the hypothesis that longer fixation to distress pictures would be shown by adolescents experiencing secure parent–adolescent attachment relationship was not supported. Complete results are available in Table 2.

**Table 2:** Associations between visual attention and aspects of parent–adolescent interaction on comfort pictures

Dimensions	Visual attention on comfort pictures	
	Fixation time	Entry time
*Dyadic*		
Collaborative communication	0.27	0.21
Warmth	0.36*	− 0.01
*Parent*		
Validation of the adolescent’s voice	0.05	0.07
Hostile/punitive	− 0.10	− 0.04
Role-confusion	− 0.32*	0.16
Odd/disorientation	− 0.11	− 0.07
*Adolescent*		
Respectful spontaneity	0.23	0.17
Hostile/punitive	− 0.14	− 0.08
Role confusion	− 0.10	0.24
Odd/disorientation	− 0.37*	− 0.18

N = 39, rho statistic is shown in table

*p < 0.05

#### Insecure/disorganized parent–adolescent interaction dimensions

We tentatively hypothesized that negative parental dimensions of hostility, role confusion, and disorientation would be associated with the adolescent’s shorter latency of SCR and shorter gaze fixation time to distress pictures. However, in contrast to secure dimensions of interaction, no significant correlations were found between insecure/disorganized parent–adolescent interaction dimensions (hostile/punitive, role-confused, odd /disoriented behaviors) and SCR in relation to distress pictures. Complete results are presented in Table 1.

Similar to results regarding secure dimensions, no significant correlations were found between insecure/disorganized parent–adolescent interaction dimensions and shorter adolescent fixation time to distress pictures. Complete results are presented in Table 2.

### Comfort picture parameters in relation to parent–adolescent interaction dimensions

#### Secure parent–adolescent interaction dimensions

We expected that the dimensions of secure interaction would be associated with higher amplitude of SCR and longer gaze fixation time to comfort pictures with a first gaze orientation on comfort pictures. Consistent with expectations, both dyadic warmth (*rho* = 0.53, *p* = 0.004) and adolescent’s respectful spontaneity (*rho* = 0.41, *p* = 0.03) were positively correlated with SCR amplitude. Additionally, warmth was negatively correlated with SCR latency (*rho* = − 0.39, *p* = 0.04). These results indicate that adolescents experiencing warmth in interaction with their parents showed a quicker and larger SCR to comfort pictures.

Also consistent with expectations, Spearman correlation analyses revealed a significant positive correlation between dyadic warmth and fixation time on comfort pictures *(rho* = 0.36, *p* = 0.03), indicating that adolescents whose parental interactions were high in warmth spent more time looking at comfort pictures. However, contrary to expectations, there were no significant associations found between secure dimensions of parent–adolescent interaction and first gaze entry time on comfort pictures.

#### Insecure/disorganized parent–adolescent interaction dimensions

Based on Syzmanska’s et al.’s [73] findings regarding response patterns of avoidant adolescents, we tentatively hypothesized that, to the comfort pictures, insecure/disorganized dimensions of parent–adolescent interaction would be associated with shorter gaze entry time to comfort pictures, accompanied by shorter overall fixation time to comfort pictures. Complete results are presented in Table 2.

Consistent with this hypothesis, a significant negative correlation was found between parent role-confusion and fixation time on comfort pictures (*rho* = − 0.32, *p* = 0.05), indicating that adolescents whose parents exhibited a higher level of role-confusion in interaction looked at comfort pictures for a significantly shorter period of time,; and also a significant negative correlation between adolescent’s odd/ disoriented behavior in the presence of the parent during the conflict discussion and shorter fixation times to the comfort picture. No significant associations were found between entry time on comfort pictures and insecure dimensions of parent–adolescent interaction. Complete results are presented in Table 2.

## Discussion

There were two major findings of the study. First, adolescents whose interactions with parents were high on secure dimensions (i.e., dyadic collaboration and warmth, parental validation of the adolescent’s voice, and adolescent’s respectful spontaneity) had a distinctive pattern of autonomic and gaze responses to distress and comfort pictures. Second, adolescents whose interactions with parents were high on insecure/disorganized dimensions did not show a similar pattern of response to distress and comfort stimuli. Both of these aspects of the findings are considered in detail below.

Secure dimensions of adolescent–parent interaction were associated a pattern of response that included both gaze parameters and aspects of autonomic arousa. Specifically, when exposed to distress pictures, adolescents who experienced more secure interactions showed a higher amplitude of SCR. This higher amplitude suggests that attachment-related distress is more salient for adolescents experiencing secure relationships. Higher amplitude of SCR was also observed in response to comfort pictures among adolescents experiencing more secure interactions. This pattern of findings seems to indicate a stronger physiological emotional response of these adolescents to both distress and comfort. This pattern is consistent with the view that secure individuals are more open to emotional experience, both negative and positive. For example, one important characteristic of Autonomous-Secure stances on the Adult Attachment Interview (AAI) is that these individuals are open to acknowledging and reflecting on both positive and negative aspects of their childhood experiences [93]. The current results point to a similar openness among adolescents experiencing warm and collaborative communication with their parents, openness to both emotions of distress and comfort.

Dimensions of security in interaction were also related to specific gaze patterns. In response to comfort pictures, adolescents who experienced more warmth in the interaction with their parents looked longer at the comfort pictures. These results are consistent with previous literature showing that maternal support during stress was associated with the child preferring to process attachment-related information [84]. In adolescents, maternal supportiveness and the ability to maintain relatedness during a conflict discussion have been closely linked to the adolescent’s attachment security [57].This result is also consistent with studies showing that less parental warmth and support during discussion of emotional situations was associated with more anxious children [94, 95]. In studies of adolescents, collaborative parent–adolescent communication was found to be a protective factor in relation to a range of maladaptive outcomes, such as externalizing behaviors, depression, dissociation, and abusive romantic relationships [25, 77, 81]. Our results add to the existing literature by finding links between caregiver warmth and valuing and adolescent behavioral and autonomic responses to attachment-themed pictures.

In addition, these results regarding collaboration and warmth appear coherent with Thompson’s claim [96] that the experience of security is based not on the denial of negative affect but on the ability to tolerate negative affects temporarily in order to achieve mastery over threatening or frustrating situations. Adolescents who are comfortable experiencing and expressing both positive and negative feelings should feel more confident in their ability to face relational distress and alleviate it.

In contrast, adolescents experiencing hostile, role-confused, or disoriented interaction with their parents did not show this pattern of response. Indeed, clear patterns of autonomic and/or gaze response did not occur in relation to any of the insecure/disorganized dimensions. We had tentatively hypothesized that these adolescents might show patterns similar to those shown by avoidant adolescents in the Szymansha et al. [73] study, characterized by quicker autonomic response to distress pictures, but shorter fixation times to both distress and comfort pictures. This deactivation of attention to arousing stimuli is consistent with the avoidance of attachment-related cues by avoidant individuals in the larger literature [23, 97].

Given that the GPACS assessment focusses more on dimensions of interaction associated with forms of disorganization, it is perhaps not surprising that consistent patterns of behavioral and autonomic responding did not emerge, in constrast to our results with secure adolescents and Szymanska et al.’s [73] results with avoidant adolescents. Disorganized individuals, in general, are found to exhibit contradictory and confused behaviors when feeling vulnerable, whether in infancy in relation to the caregiver, or on the AAI in relation to themes of loss or trauma [30]. More work is needed to understand how to characterize the ways that adolescents in more hostile, role-confused, or disoriented relationships regulate attention and arousal in relation to attachment-themed stinmuli.

Notably, adolescents of parents who were more role-confused in interaction spent less time looking at comfort pictures than other adolescents. The parental role-confusion scale assesses the extent to which the parent fails to maintain a parental stance by failing to structure the interaction, failing to contribute to the task goals (discuss the conflict), remaining excessively self-focused (e.g., prioritizing his/her own needs over those of the adolescent) or treating the adolescent like a peer or a romantic partner. As the attachment relationship is geared toward regulating the negative affect of the child, negative affects remain unregulated by the attachment figure in a role-confused dyad. In relation to the current findings, both the enhanced fixation time shown by secure adolescents to comfort pictures and the reduced fixation time shown by adolescents experiencing more role-confused relationships, taken together, suggest that adolescents in role-confused relationships may have the least experience or expectation of comfort compared to other adolescents in the study. However, further work replicating this finding in relation to other paradigms involving response to comfort will be needed to know whether a more generalized interpretation is warranted. Nonetheless, it is clear from other work that role-confusion within the parent–child relationship is closely linked with dysregulation of affects and increased involvement in risk behaviors [81, 98, 99]. For example, Kobak showed that male adolescents in role-confused dyads reported increased involvement in risky behaviors, including unprotected sexual activity and substance use problems, from age 13 to 15 [25]. Lyons-Ruth et al. [79] similarly found that role-confusion was an important correlate of borderline features and suicidality.

Finally, adolescents who themselves exhibited more odd, out-of context, disoriented behavior in the presence of the parent during the conflict discussion also exhibited significantly shorter fixation times to the comfort pictures. There have been fewer studies related to adolescent odd, out-of-context, disoriented behavior. However, the existing studies suggest that this dimension captures behavior related to more severe histories of maltreatment [79, 81], as well as to current suicidality and borderline personality disorder [79, 82]. Thus, while current results need to be replicated in additional samples, reduced fixation time to comfort pictures may be one indicator of more disturbed parent–adolescent relationships.

This study adds an important new dimension to the understanding of how attachment patterns may be related to emotion regulation, because few studies have assessed physiological reactivity to attachment-themed stimuli in adolescents in relation to observed quality of parent–adolescent interaction. Instead, most studies have focused on the adolescent’s physiological reactivity in relation to adolescent attachment classifications on the AAI or the Attachment Styles Interview [75], with contradictory results [61, 73, 100, 101]. Directly observing adolescent–parent interaction allows a much more nuanced assessment of the quality of the relationship, because the parent is also being observed as part of the assessment, while interview measures do not give access to parenting quality directly.

Several methodological limitations of this study should be considered in relation to directions for future work. Small sample size, the limited number of fathers and boys who participated, and the low-risk nature of our sample limited the generalizability of our results. Replication of this design in larger and more diverse samples, including high-risk and clinical populations would be important in the future. A larger sample would also allow more complex analyses, controlling for a range of potentially confounding variables. A larger sample size would also allow for the consideration of age in the analyses and thus the assessment of possible developmental changes in the patterns of findings seen here [41, 43]. A further limitation is that organized insecure dimensions of interaction (dismissing, preoccupied) were not assessed in this version of the GPACS. Since this study was completed, scales for avoidant and preoccupied aspects of interaction have been developed. Thus, an important area for future research would be to assess insecure-organized dimensions of interaction more explicitly, since these aspects of interaction are more prevalent in low-risk populations. Additionally, because this is a cross-sectional study, no relation of causality can be established between aspects of parent–adolescent interaction and parameters of gaze or arousal. Finally, only one study to date has assessed longitudinal predictors of GPACS dimensions [25]. Such longitudinal studies would contribute to efforts to establish direction of effect.

Despite these limitations, this study is the first to use observational assessment tools and dynamic psychobiological measures to further characterize the link between attachment relationships and emotion regulation processes in a low-risk, non-clinical sample of adolescents and their parents. These findings should catalyze other research to use novel methodologies to better understand how relationships are linked to attentional processes and arousal regulation.

In conclusion, the current study adds to the increasing body of evidence supporting a role for the quality of parent–adolescent interaction in the adolescent’s ability to regulate emotions in adaptive or maladaptive ways. Our findings indicate that higher physiological responses to both distress and comfort stimuli, which represent essential attachment-related experiences, are associated with observed dimensions of secure parent–adolescent interaction and thus are likely to represent physiological and behavioral indicators of more adaptive emotion regulation. Conversely, adolescents experiencing role confusion, parental hostility, or odd, out- of- context behaviors in interaction do not show similar patterns of response to stimuli associated with distress and comfort. Clinically, these results add to the body of data supporting the pervasive influence of open, balanced, and collaborative parent–adolescent relationships on behavioral and physiological functioning. Future work should assess the potential for developing more effective supportive interventions to help parents maintain a parental role, protect the adolescent during vulnerable moments, and communicate in more balanced and valuing ways with their adolescents.

## Data Availability

The datasets generated and/or analyzed during the current study are not publicly available because the participants did not permit to share their data individually rather than an aggregate report, but are available from the corresponding author on reasonable request.

## Abbreviations

AAI: Adult attachment interview
ER: Emotion regulation
GPACS: Goal corrected partnership in adolescence coding system
SCR: Skin conductance response
VAREA: Visual/Autonomic Regulation of Emotions Assessment
